# Ivermectin Inhibits Growth of *Chlamydia trachomatis* in Epithelial Cells

**DOI:** 10.1371/journal.pone.0048456

**Published:** 2012-10-30

**Authors:** Matthew A. Pettengill, Verissa W. Lam, Ikechukwu Ollawa, Camila Marques-da-Silva, David M. Ojcius

**Affiliations:** 1 Molecular Cell Biology, Health Sciences Research Institute, University of California Merced, Merced, California, United States of America; 2 Children’s Hospital Boston, Harvard Medical School, Boston, Massachusetts, United States of America; 3 Biophysics Institute Carlos Chagas Filho, Federal University of Rio de Janeiro, Rio de Janeiro, Brazil; University Paris Sud, France

## Abstract

Ivermectin is currently approved for treatment of both clinical and veterinary infections by nematodes, including *Onchocerca cervicalis* in horses and *Onchocerca volvulus* in humans. However, ivermectin has never been shown to be effective against bacterial pathogens. Here we show that ivermectin also inhibits infection of epithelial cells by the bacterial pathogen, *Chlamydia trachomatis*, at doses that could be envisioned clinically for sexually-transmitted or ocular infections by *Chlamydia*.

## Introduction

Avermectins are macrocyclic lactone derivatives which are produced during fermentation of *Streptomyces avermectinius*, a species of actinomycete isolated from soil samples in Japan. Eight avermectins are produced by *S. avermectinius* (A_1a_, A_1b_, A_2a_, A_2b_, B_1a_, B_1b_, B_2a_, and B_2b_). The A series molecules contain a 5′-methoxyl group, while the B series contain a 5′-hydroxyl group and have more potent anti-parasitic activity [1], [2], [3]. Improved efficacy as a broad-spectrum anti-parasitic was achieved for the B1 compounds by selective hydrogenation utilizing Wilkinson’s catalyst (RhCl(PPh_3_)_3_), with the resultant product (22,23-dihydroavermectin B1) being given the name ivermectin [4]. Following considerable success as an anti-helminthic agent in veterinary practice, including use for treatment of *Onchocerca cervicalis* in horses, ivermectin entered clinical trials for use in humans against *Onchocerca volvulus*, a nematode which causes onchocerciasis (“river blindness”) [5]. Efficacious use in humans has extended to lymphatic filariasis [6], head-lice infestation [7], and scabies [8]. However, avermectins have not been previously described to have anti-bacterial activity [5].

Here we demonstrate that ivermectin inhibits growth of *Chlamydia trachomatis* during infection of human cervical epithelial cells, suggesting that avermectins may have previously undescribed anti-bacterial activity against pathogenic obligate intracellular bacteria, potentially via indirect effects on the host-cell.

Chlamydiae are obligate intracellular bacteria which mature through a unique biphasic developmental cycle, infecting as metabolically inert elementary bodies (EBs) and maturing into metabolically active but non-infectious reticulate bodies (RBs), which proliferate before condensing into infectious EBs to complete the cycle [9], [10], [11]. *C. trachomatis* strains, which are the leading cause of bacterial sexually transmitted disease and first cause of preventable blindness [12], primarily infect mucosal epithelial cells.

## Results and Discussion

**Figure 1:**
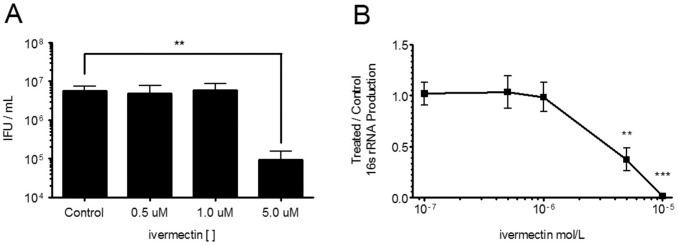
Ivermectin inhibits chlamydial infection of epithelial cells. (A) HeLa cells were infected with *C. trachomatis* serovar L2 at an MOI of 1, followed by treatment with ivermectin at the indicated concentrations at 1 hour post infection (hpi). Samples were harvested at 24 hpi for quantification of reinfectious yield (IFU/ml) on new HeLa cell monolayers utilizing fluorescent microscopy and anti-*C. trachomatis* antibodies. The values show means plus standard deviation of three independent experiments. (n = 3,**, P = 0.0059). (B) Total RNA was harvested at 24 hpi for quantification of chlamydial 16s rRNA. The values shown are relative to control values for each experiment, and are means and standard deviations of 3 independent experiments (n = 3, **, P = 0.0034, ***, P<0.001, compared to 100 nM condition). Two-tailed unpaired t tests were performed using GraphPad Prism version 5.0b for Mac.

**Figure 2:**
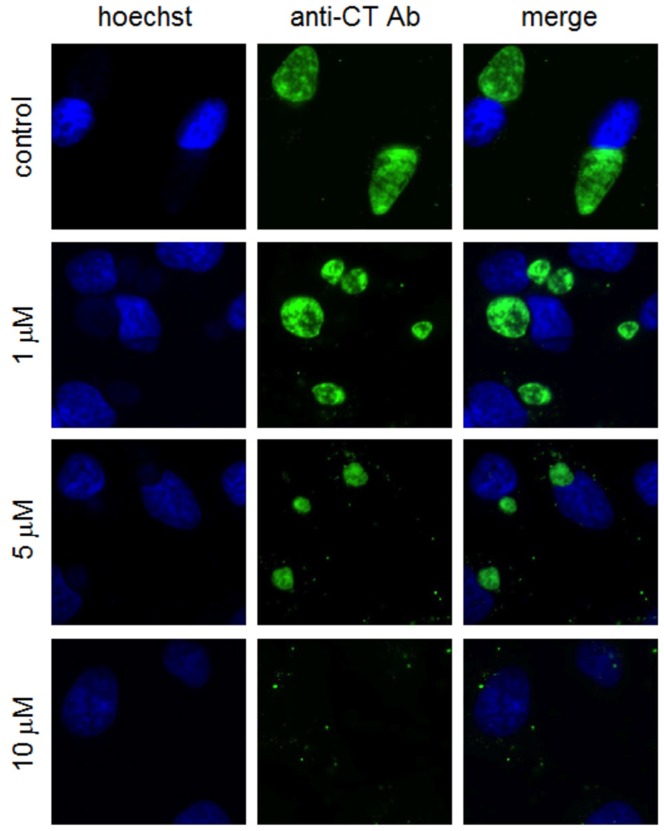
Ivermectin inhibits the development of chlamydial inclusions in epithelial cells. HeLa cells grown on glass coverslips were infected with C. trachomatis serovar L2 at an MOI of 1, followed by treatment with ivermectin at the indicated concentrations at 1 hour post infection (hpi). At 24 hpi, cells were fixed with ice cold methanol for 10 minutes, followed by staining with *C. trachomatis* genus antibodies (Argene) and Hoechst (Sigma), and observed on a widefield fluorescence microscope (Leica).

Five µM ivermectin added one hour post infection (hpi) significantly inhibited the production of infectious EB (Figure 1A) and chlamydial 16s rRNA accumulation (Figure 1B), as evaluated by harvesting infected HeLa cultures and infecting fresh HeLa monolayers followed by fluorescent microscopy to determine infectious units per mL (IFU/ml, Figure 1A), or by RNA extraction of infected HeLa cultures followed by cDNA synthesis and qPCR for *C. trachomatis* 16s rRNA (Figure 1B). Treatment with 1 µM ivermectin at 1 hpi modestly decreased the size of inclusions, while inclusions in cells treated with 5 µM ivermectin had significantly reduced dimensions (Figure 2). Treatment with 10 µM ivermectin completely inhibited inclusion development. Of note, 5 µM ivermectin did not reduce the number of cells in which inclusions were apparent, suggesting that the effect of ivermectin was not related to uptake of bacteria into host cells.

**Figure 3:**
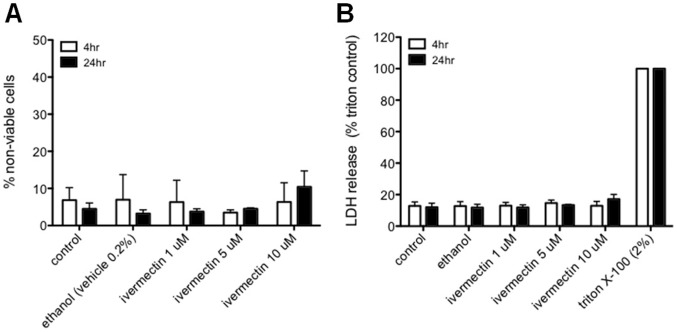
Ivermectin is not cytotoxic to epithelial cells. (A) HeLa cells were cultured in the presence of the indicated concentrations of ivermectin or vehicle (0.2% ethanol), supernatants were then centrifuged at 500 g for 5 minutes, and adherent cells were removed with trypsin/EDTA and gently recombined with supernatant pellets. Cell viability was determined by trypan blue exclusion, evaluated using a hemocytometer. (B) Supernatants from the cultures described in panel A, and from wells treated with 2% Triton X-100 30 minutes prior to collection time points, were combined and evaluated for LDH activity using a Roche Cytotoxicity Detection Kit per the manufacturer’s instructions. Shown are means plus standard deviations for 3 independent experiments.

To exclude the possibility that ivermectin may be inhibiting infection through an indirect cytotoxic effect on the host cells, we verified that treatment of HeLa cells with up to 10 mM ivermectin for 4 to 24 hours did not cause increased cell death (Figure 3A) or lactate dehydrogenase (LDH) release (Figure 3B).

For use in humans as an anti-helminthic, ivermectin is typically received orally at a dose of 150 µg/kg body weight, and peak plasma concentrations from such dosage reach around 60 nM [13]. Ivermectin has also been used and well-tolerated at 500 µg/kg body weight via topical administration in veterinary applications [14], which could potentially be an attractive mode of use if it is found to be effective against intracellular bacterial pathogens which primarily infect readily accessible mucosal surfaces. Ivermectin targets glutamate-gated chloride channels in nerve and muscle cells and gamma-aminobutyric acid (GABA) related chloride channels of invertebrates [5], as well as mammalian GABA receptors [15].

Ivermectin has also been reported to interact with the purinergic receptor, P2X_4_ [16], which can be stimulated by low (micromolar) concentrations of ATP; and we have observed that stimulation of *C. trachomatis*-infected epithelial cells with micromolar concentrations of ATP leads to chlamydial growth inhibition [17]. However, addition of apyrase (2.5 U/ml) to cells immediately prior to ATP addition prevents ATP-mediated chlamydial growth inhibition (unpublished data), but does not modify the impact of ivermectin on chlamydial growth (1 or 2.5 U/ml apyrase, 5 µM ivermectin, N = 3; data not shown). These results suggest that ivermectin does not inhibit infection through P2X_4_ ligation.

We have here demonstrated that ivermectin inhibits *C. trachomatis* infection in epithelial cells. While the concentrations of ivermectin necessary for this inhibitory action in vitro are higher than what is achieved distal to absorption sites in current human therapy, topical application may allow therapeutic use of ivermectin against sexually-transmitted infection, or against eye infection with ocular strains of *C. trachomatis*. Additionally, as the target of this particular activity in human cells has yet to be identified, other avermectins or structurally modified avermectin molecules may have greater potency. As it seems that ivermectin mediates this response through interaction with a host cell target, potential efficacy against other obligate intracellular bacteria or parasites is worthy of exploration.

## Materials and Methods

### Cells and Materials

The epithelial cell line HeLa 229 (American Type Culture Collection, Manassas, VA) was used to model infection with the LGV/L2 strain of *C. trachomatis* (obtained from Dr. Roger Rank, University of Arkansas, Little Rock, AR). HeLa cells were cultured in a humidified incubator at 37 °C with 5% CO_2_ in Dulbecco’s modified Eagle medium (DMEM:F-12, Invitrogen) supplemented with 10% heat-inactivated fetal calf serum. Apyrase and Hoechst were purchased from Sigma (St. Louis, MO), and antibodies against the *C. trachomatis* genus antibodies were from Argene (Sherley, NY).

### Preparation and Use of Ivermectin

A concentrated stock of ivermectin (Sigma, St. Louis, MO) was solubilized in ethanol, and dilutions were also prepared in ethanol. Control wells received the same concentration of ethanol (0.2%) as treated wells, which did not influence chlamydial infection relative to control wells without ethanol (data not shown).

### Chlamydial Infection and Analysis

HeLa cells growing at 70% confluence in tissue culture plates (Costar) were infected at a multiplicity of infection (MOI) of 1.0, with treatments at the indicated times [18]. Infection was assayed by fluorescence microscopy and qPCR for *C. trachomatis* 16s rRNA, using protocols and primers previously described [18]. To analyze inclusion size and morphology by fluorescent microscopy, HeLa cells were grown on glass coverslips, and after the indicated experimental conditions were fixed with ice cold methanol for 10 minutes. Cells were stained with *C. trachomatis* genus antibodies from Argene (North Massapequa, NY) and Hoechst (Sigma), and observed on a widefield fluorescence microscope (Leica, Deerfield, IL).

Statistical analysis was performed using GraphPad Prism version 5.0b for Mac (GraphPad Software, San Diego, CA).

## References

[1] Burg RW, Miller BM, Baker EE, Birnbaum J, Currie SA, et al (1979) Avermectins, new family of potent anthelmintic agents: producing organism and fermentation. Antimicrob Agents Chemother 15: 361–367. 464561 10.1128/aac.15.3.361 PMC352666

[2] Egerton JR, Ostlind DA, Blair LS, Eary CH, Suhayda D, et al (1979) Avermectins, new family of potent anthelmintic agents: efficacy of the B1a component. Antimicrob Agents Chemother 15: 372–378. 464563 10.1128/aac.15.3.372 PMC352668

[3] Miller TW, Chaiet L, Cole DJ, Cole LJ, Flor JE, et al (1979) Avermectins, new family of potent anthelmintic agents: isolation and chromatographic properties. Antimicrob Agents Chemother 15: 368–371. 464562 10.1128/aac.15.3.368 PMC352667

[4] Chabala JC, Mrozik H, Tolman RL, Eskola P, Lusi A, et al (1980) Ivermectin, a new broad-spectrum antiparasitic agent. J Med Chem 23: 1134–1136. 6893469 10.1021/jm00184a014

[5] Omura S, Crump A (2004) The life and times of ivermectin - a success story. Nat Rev Microbiol 2: 984–989. 15550944 10.1038/nrmicro1048

[6] Taylor MJ, Hoerauf A, Bockarie M (2010) Lymphatic filariasis and onchocerciasis. Lancet 376: 1175–1185. 20739055 10.1016/S0140-6736(10)60586-7

[7] Chosidow O, Giraudeau B, Cottrell J, Izri A, Hofmann R, et al (2010) Oral ivermectin versus malathion lotion for difficult-to-treat head lice. N Engl J Med 362: 896–905. 20220184 10.1056/NEJMoa0905471

[8] Currie BJ, McCarthy JS (2010) Permethrin and ivermectin for scabies. N Engl J Med 362: 717–725. 20181973 10.1056/NEJMct0910329

[9] Bavoil PM, Hsia R-C, Ojcius DM (2000) Closing in on *Chlamydia* and its intracellular bag of tricks. Microbiol 146: 2723–2731. 10.1099/00221287-146-11-2723 11065351

[10] Fields KA, Hackstadt T (2006) The *Chlamydia* Type III Secretion System: Structure and Implications for Pathogenesis. In: Bavoli PM, Wyrick PB, editors. *Chlamydia*: Genomics and Pathogenesis. 219–233.

[11] Wyrick PB (2000) Intracellular survival by *Chlamydia* . Cell Microbiol 2: 275–282. 11207584 10.1046/j.1462-5822.2000.00059.x

[12] Belland R, Ojcius DM, Byrne GI (2004) Chlamydia. Nat Rev Microbiol 2: 530–531. 15248311 10.1038/nrmicro931

[13] Baraka OZ, Mahmoud BM, Marschke CK, Geary TG, Homeida MM, et al (1996) Ivermectin distribution in the plasma and tissues of patients infected with Onchocerca volvulus. Eur J Clin Pharmacol 50: 407–410. 8839664 10.1007/s002280050131

[14] Gokbulut C, Cirak VY, Senlik B, Aksit D, Durmaz M, et al (2010) Comparative plasma disposition, bioavailability and efficacy of ivermectin following oral and pour-on administrations in horses. Vet Parasitol 170: 120–126. 20181429 10.1016/j.vetpar.2010.01.041

[15] Adelsberger H, Lepier A, Dudel J (2000) Activation of rat recombinant alpha(1)beta(2)gamma(2S) GABA(A) receptor by the insecticide ivermectin. Eur J Pharmacol 394: 163–170. 10771281 10.1016/s0014-2999(00)00164-3

[16] North RA (2002) Molecular physiology of P2X receptors. Physiol Rev 82: 1013–1067. 12270951 10.1152/physrev.00015.2002

[17] Pettengill MA, Marques-da-Silva C, Avila ML, d’Arc Dos Santos Oliveira S, Lam VW, et al. (2012) Inhibition of *Chlamydia trachomatis* Infection in Epithelial Cells Due to Stimulation of P2X4 Receptors. Infec Immun in press. 10.1128/IAI.00441-12 PMC3497399 22988022

[18] Pettengill MA, Lam VW, Ojcius DM (2009) The danger signal adenosine induces persistence of chlamydial infection through stimulation of A2b receptors. PLoS One 4: e8299. 20011598 10.1371/journal.pone.0008299 PMC2788228

